# Supplementary material for: Structural and kinetic analysis of the COP9-Signalosome activation and the cullin-RING ubiquitin ligase deneddylation cycle
Source: eLife. 2016 Mar 31;5:e12102. doi: 10.7554/eLife.12102 (PMC4878873; doi:10.7554/eLife.12102)
Supplement: Related texts

# Article commentary: Decision letter

**DOI:** 10.7554/eLife.12102.030

**Affiliations:** Harvard Medical School, United States

In the interests of transparency, eLife includes the editorial decision letter and accompanying author responses. A lightly edited version of the letter sent to the authors after peer review is shown, indicating the most substantive concerns; minor comments are not usually included.

Thank you for submitting your article "Structural and kinetic analysis of CSN activation and the cullin-RING ubiquitin ligase deneddylation cycle" for consideration by *eLife*. Your article has been favorably evaluated by Ivan Dikic (Senior editor) and three reviewers, one of whom, Wade Harper, is a member of our Board of Reviewing Editors.

The reviewers have discussed the reviews with one another and the Reviewing Editor has drafted this decision to help you prepare a revised submission.

Summary:

This paper provides a structural and kinetic analysis of the Cop9/Signalosome complex (CSN). The authors provide a structure of the 8 subunit CSN complex bound to a CRL. They investigate the mechanism of action using kinetic approaches and describe an "E-swap" model the catalytic mechanism. This provides the first systematic analysis of CSB kinetics.

Essential revisions:

1) One reviewer felt that, from the data available, it was unclear whether the fit of Nedd8 and Rbx1 in the EM structure was as good as suggested in the text. For example, the statement "…allowed us to precisely describe the movements…" may be overstating the case somewhat. So the text should be toned down and it should be made clear what the limits of resolution and fitting are in this structure, given that it is only at ~7A.

2) A second major comment concerns how the kinetic studies were performed in order to obtain *K_d_* values. The kinetic binding studies were performed with a fixed concentration of Cul1 at 30 nM. However, the *K_d_* values are in the single nM range, raising questions about the accuracy of the data due to the difficulty in getting accurate values when the protein concentration is higher than *K_d_* due to the steepness of the equilibrium binding graph (see Pollard T.D. 2010, Molecular Biology of the Cell v.21 p4061-4067). For example, the plot of equilibrium binding of WT CSN with Cul1/Rbx1 reports a *K_d_* of 310 nM, which can be easily identified by inspection of the graph in Figure 2B. In contrast the *K_d_* values when *K_d_* < 30 nM cannot be inferred from their respective graphs. The problem becomes apparent if one were to plot theoretical graphs of equilibrium binding of *K_d_*s of 1.6 nM, 5 nM, and 10 nM where the fixed protein concentration is 30 nM. These graphs would almost lie entirely on top of each other. The data in the paper suggests this problem as the 5 nM *K_d_* measured for CSN^5H138A^ in Figure 3—figure supplement 1K doesn't match the previously stated value of 1.6 nM or 10 nM. The differences in dissociation constants are large enough between WT and mutant CSN that the general message of the paper is very likely correct, but it would probably be best if key measurements could be attempted with lower CUL1 levels. However, if this isn't possible for technical reasons, you could perhaps emphasize the *K_d_*s in the low nanomolar range (those higher than the reported limit of 1.5 nM). It was also suggested that you may want to describe the limits of the calculated *K_d_* of 4 nM reported in Figure 3—figure supplement 1C for the SCF Fbxw7-CSN5H138A complexes as the error bars are large.

# Reply: Author response

**DOI:** 10.7554/eLife.12102.031

Essential revisions:

*1) One reviewer felt that, from the data available, it was unclear whether the fit of Nedd8 and Rbx1 in the EM structure was as good as suggested in the text. For example, the statement "…allowed us to precisely describe the movements…" may be overstating the case somewhat. So the text should be toned down and it should be made clear what the limits of resolution and fitting are in this structure, given that it is only at ~7A.*

We appreciate the cautioning remark and have re-worded the text in the second paragraph of the Results section, as well as the ends of the respective paragraphs describing the docking of the RING domain and Nedd8 and the WHB domain of Cul1 (subsection “Structural insights from cryo EM and single particle analysis of a CSN-SCF-Nedd8Skp2/Cks1 complex”). We have thus made it clear that the current resolution does not permit the precise fitting of small subunits like Rbx1 or Nedd8. Nevertheless, the primary advance over previous structural work is that we could position these small subunits/domains relative to CSN, and consequently formulate hypotheses, tested by mutants predicted from the structure.

*2) A second major comment concerns how the kinetic studies were performed in order to obtain K_d_ values. The kinetic binding studies were performed with a fixed concentration of Cul1 at 30 nM. However, the K_d_ values are in the single nM range, raising questions about the accuracy of the data due to the difficulty in getting accurate values when the protein concentration is higher than K_d_ due to the steepness of the equilibrium binding graph* (*see Pollard T.D. 2010, Molecular Biology of the Cell v.21 p4061-4067*)*. For example, the plot of equilibrium binding of WT CSN with Cul1/Rbx1 reports a K_d_ of 310 nM, which can be easily identified by inspection of the graph in Figure 2B. In contrast the K_d_ values when K_d_ < 30 nM cannot be inferred from their respective graphs. The problem becomes apparent if one were to plot theoretical graphs of equilibrium binding of K_d_s of 1.6 nM, 5 nM, and 10 nM where the fixed protein concentration is 30 nM. These graphs would almost lie entirely on top of each other.*

We agree with the concern of the reviewer, and we are also familiar with the Pollard article that the reviewer cites. We settled on a Cul1^d^ concentration of 30 nM because it is the lowest concentration at which we can consistently get reliable experimental measurements. While it is possible, when everything works perfectly, to drop the Cul1^d^ concentration somewhat below 30 nM, under no circumstances can we work at single digit nM concentrations. We thus cannot make the simplifying assumption that [CSN]_free_ = [CSN]_tot_ and resorted to the standard practice of using a quadratic equation to estimate the *K_d_*. While it is more challenging (and the results are more prone to error) to measure a *K_d_* below the concentration of the species that is held fixed, it is not impossible. Author response image 1 shows theoretical curves calculated for a ligand that binds a receptor (held at 30 nM) with a *K_d_* of, 10 nM, 5 nM and 2 nM. As can be seen, distinguishing between 1.6 and 5 nM is difficult, but the shape of the binding curve we see appears to be consistent with a *K_d_* well below 10 nM. We feel the most appropriate response given the technical challenges is to state very clearly in the text the limitations of measuring a *K_d_* that is below the concentration of the species that is held constant. Accordingly, we now state the following: “Note that the estimated *K_d_* falls well below the fixed concentration of Cul1^d^-N8/Rbx1 used in the assay. This introduces greater uncertainty into our estimate but nevertheless our data suggest that the binding of substrate to CSN^5H138A^ is tight (≤5 nM; see Materials and methods for further discussion of this matter)”.

**Author response image 1.** **DOI:** http://dx.doi.org/10.7554/eLife.12102.027

In the Methods we state: “It should be noted that several of the *K_d_* values reported for CSN binding to Cul1^d^-N8/Rbx1 or Cul1^d^/Rbx1 are below the concentration of the dansylated ligand (30 nM). While this is not optimal, we found that 30 nM was the lowest concentration that consistently yielded highly reproducible results. A *K_d_* was estimated by the standard approach of using a quadratic equation as described above. The estimated *K_d_* is very sensitive to the density of data points at the inflection point of the curve, and thus these estimates can be more prone to error. Nevertheless, different investigators in Zurich and Pasadena have consistently obtained an estimate ranging from 1.6 to 5 nM for binding of CSN^5H138A^ to Cul1^d^-N8/Rbx1 and of 9 to 13 nM for binding to CSN^5H138A^ to Cul1^d^/Rbx1, using different protein preparations.”

The data in the paper suggests this problem as the 5 nM K_d_ measured for CSN^5H138A^ in Figure 3—figure supplement 1K doesn't match the previously stated value of 1.6 nM or 10 nM.

We are aware of this, but we wish to emphasize that the point of Figure 3—figure supplement 1K was to show that deletion of the N-terminal domain of Csn2 had a massive effect on substrate binding. The CSN^5H138A^ was included as the control for the experiment. Because we knew the difference would be huge, we collected a smaller number of data points for CSN^5H138A^ in this experiment. Consequentially the estimate in Figure 3—figure supplement 1K is less accurate. This does not in any way affect the interpretation of this experiment.

*The differences in dissociation constants are large enough between WT and mutant CSN that the general message of the paper is very likely correct, but it would probably be best if key measurements could be attempted with lower CUL1 levels. However, if this isn't possible for technical reasons, you could perhaps emphasize the K_d_s in the low nanomolar range (those higher than the reported limit of 1.5 nM).*

We hope the editor agrees that the additional text we have added to the Results and Materials and methods adequately deals with this issue.

It was also suggested that you may want to describe the limits of the calculated K_d_ of 4 nM reported in Figure 3—figure supplement 1C for the SCF Fbxw7-CSN5H138A complexes as the error bars are large.

We are aware of this problem but did not invest a great deal of effort in addressing it because we did not use Fbxw7-Skp1 in subsequent experiments. As noted above, measurements within the range of 1.6-5 nM are within the error of estimation. We now state in the legend of Figure 3—figure supplement 1C: “Note that addition of Fbxw7–Skp1 greatly increased the variability in the measurement for unknown reasons.”

To maintain some perspective here, we would like to emphasize that the binding of substrate to enzyme is exceedingly tight. Whether it is 1.6 nM or 5 nM is inconsequential. The big surprise is that it is so tight (obviously far below 30 nM). This is unexpected given that the *K_M_* is ~200 nM and the affinity for product is ~100-fold lower, despite the fact that Nedd8 itself appears to have relatively limited affinity for CSN. This extremely high affinity for substrate, the large disconnect between *K_d_* and *K_M_*, and the great drop in affinity upon cleavage of Nedd8 have important implications for physiology, as we describe in the Discussion.
